# Deficiency of C-C Chemokine Receptor 5 Suppresses Tumor Development *via* Inactivation of NF-κB and Upregulation of IL-1Ra in Melanoma Model

**DOI:** 10.1371/journal.pone.0033747

**Published:** 2012-05-02

**Authors:** Ju Kyoung Song, Mi Hee Park, Dong-Young Choi, Hwan Soo Yoo, Sang Bae Han, Do Young Yoon, Jin Tae Hong

**Affiliations:** 1 College of Pharmacy and Medical Research Center, Chungbuk National University, Cheongju, Chungbuk, Republic of Korea; 2 Department of Bioscience and Biotechnology, Bio/Molecular Informatics Center, Konkuk University, Seoul, Republic of Korea; Sun Yat-sen University Medical School, China

## Abstract

To evaluate the relevance of C-C chemokine receptor type 5 (CCR5) expression and tumor development, we compared melanoma growth in CCR5 knockout (CCR5^−/−^) mice and wild type (CCR5^+/+^) mice. CCR5^−/−^ mice showed reduced tumor volume, tumor weight, and increased survival rate when compared to CCR5^+/+^ mice. We investigated the activation of NF-κB since it is an implicated transcription factor in the regulation of genes involving cell growth, apoptosis, and tumor growth. Significant inhibition of DNA binding activity of NF-κB, and translocation of p50 and p65 into the nucleus through the inhibition of phosphorylation of IκB was found in the melanoma tissues of CCR5^−/−^ mice compared to melanoma tissues of CCR5^+/+^ mice. NF-κB target apoptotic protein expression, such as cleaved caspase-3, cleaved PARP, and Bax, was elevated, whereas the survival protein expression levels, such as Bcl-2, C-IAP1, was decreased in the melanoma tissues of CCR5^−/−^ mice. Interestingly, we found that the level of IL-1Ra, a tumor growth suppressive cytokine, was significantly elevated in tumor tissue and spleen of CCR5^−/−^ mice compared to the level in CCR5^+/+^ mice. Moreover, infiltration of CD8^+^ cytotoxic T cell and CD57^+^ natural killer cells was significantly increased in melanoma tumor and spleen tissue of CCR5^−/−^ mice compared to that of CCR5^+/+^ mice. Therefore, these results showed that CCR5 deficiency caused apoptotic cell death of melanoma through inhibition of NF-κB and upregulation of IL-1Ra.

## Introduction

Chemokines are small soluble molecules that are best known for their potent ability to induce cancer cell growth by inflammation. Many types of cancer cells express chemokines and chemokine receptors [1]. The accumulated evidence indicates that the chemokine (C-C motif) ligand 5 (CCL5) and C-C chemokine receptor (CCR5), which are potent chemotactic factors for inflammatory cells, may be significantly involved in the proliferation and metastasis of several cancers. Intermediate and strong CCR5 expression is significantly associated with nonmetastatic development of colorectal cancer [2], melanoma [3], hepatoma [4], glioblastoma, Hodgkin lymphoma [5], as well as oral and prostate cancer cells [6]. Local production of the CCL5 is also important in the progression of breast cancer [7], and also correlates with poor prognosis [8]. Meanwhile, CCR5 disruption has been demonstrated to inhibit experimental tumor growth and metastasis of pancreatic cancer [9]. In addition, host absence of CCR5 potentiates the delay of tumor growth [10], and CCR5 inhibitors prevented cancer cell growth, such as prostate cancer [11], breast cancer, hepatoma cells [4], and lung cancer [12]. These data suggest that the deficiency of inflammatory chemokine receptor CCR5 may function as a suppressive receptor in cancer progression. However, more studies are required to describe how CCR5 deficiency acts in the inhibition of tumor development.

Interleukin-1 receptor antagonist (IL-1Ra), a member of the IL-1 family, is a naturally occurring cytokine that competitively blocks the IL-1 receptor [13]. Because IL-1Ra has been shown to inhibit tumor progression by promoting antitumor immune responses and by enhancing the activity of chemotherapy, numerous studies have examined the ability of IL-1Ra to block tumor progression. IL-1Ra expression was reduced in prostate [14], breast [15], and skin cancer [16]. It was also reported that endogenous IL-1Ra deficient mice developed aggressive tumors following exposure to carcinogens [17]. Meanwhile, proliferation of the melanoma cells that were transduced with IL-1Ra gene was reduced and inhibited melanoma development [18]. IL-1R blockade also reduced hepatic tumor growth [19]. Moreover, it has also been reported that IL-1Ra reduces fibrosarcoma development [20], B16 melanoma growth and metastasis [21], colon adenocarcinoma growth [22], and skin cancer development [23]. IL-1 knockout mice injected with melanoma failed to develop solid tumors [24]. It was also found that IL-1Ra, combined with temozolomide and docetaxel chemotherapy, demonstrated more significant antitumor activity against B16 melanoma cells in vivo [25]. These data indicate that IL-1Ra is important as a tumor growth suppressing cytokine.

NF-κB plays a crucial role in the suppression of apoptosis, as well as the induction of cell proliferation and inflammation, and is closely associated with cancer development [26]. Constitutive activation of NF-κB has been described in a great number of cancers including colon cancers, prostate cancers and melanoma [27], and was found to up-regulate anti-apoptotic genes and/or down regulate apoptotic gene expression [28]. Although NF-κB promotes tumor growth, it is required for the immune system to function normally [29]. Studies on c-Rel-deficient mice demonstrated that c-Rel is essential for IL-2, IL-3, GM-CSF, and IFNγ expression in T lymphocytes [30]. C-Rel-deficient mice also have a tissue-specific deficiency of various cytokines and growth factors in T cells and macrophages affecting both innate and humoral immune responses in the host [31]. Therefore, NF-κB can be specially targeted to prevent cancer cell growth by (1) directly affecting apoptotic cancer cell death, (2) stimulating tumor killing lymphocytes infiltration or (3) increasing the production of tumor killing lymphokines. Activation of NF-κB is important in the regulation of CCR-mediated tumor growth and metastasis. It was reported that inhibition of NF-κB by dehydroxymethyl-epoxyquinomicin (DHMEQ) induces cell death of primary effusion lymphoma by inhibition of CCR5 expression [32]. Activation of NF-κB also increases CCL5-mediated oral cancer motility (5) and lung metastasis [33]. Thus, it is possible that inhibition of NF-κB could be involved in CCR5 mediated tumor development.

NF-κB transcription factor binding sites are present in the promoter region of proinflammatory cytokine genes, such as IL-1alpha, IL-6, GM-CSF, and KC [34]. On the other hand, the IL-1Ra is a negative regulator of the inflammatory response. Several studies have demonstrated the ability of IL-1Ra to abrogate the proinflammatory effects of IL-1. Smith et al. suggested that the regulation of IL-1Ra gene expression is a complex event involving the interactions of three different transcription factors; NF-κB/PU.1/GA-binding protein binding site [35]. Nuclear translocation of NF-κB (p65) was significantly enhanced and prolonged in IL-1Ra-deficient mice, compared to that in wild type mice [36]. Intracellular IL-1Ra (icIL-1Ra) was found to inhibit IL-1 by blocking NF-κB signal transduction pathways in inflammatory responses [37]. Because IL-1 has been shown to be an inducer of NF-κB activation [38], we hypothesized that the expression of IL-1Ra could play an important role in the autocrine activation of transcription factors NF-κB or vice versa. Thus, the aim of this study was to evaluate the roles of NF-κB and IL-1Ra in the chemokine receptor CCR5-mediated suppression of tumor development.

## Results

### Inhibition of Tumor Development in CCR5^−/−^ Mice

**Figure 1:**
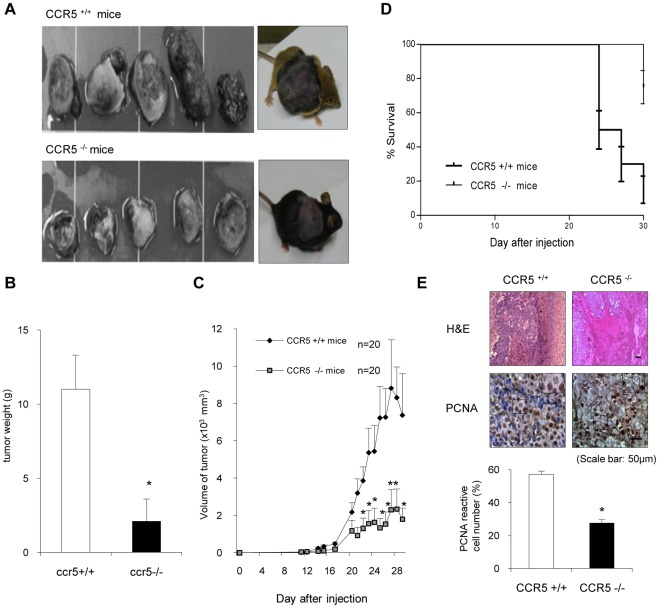
Inhibition of tumor development in CCR5^−/−^ mice. **A–C,** Tumor images, volumes, and weights. B16 melanoma cells (5×10^5^ cells/mouse) were inoculated s.c. into CCR5^+/+^ mice and CCR5^−/−^ mice (n = 20). Tumor volumes were measured every day and tumor weights were measured at study termination (Day 31). The CCR5^−/−^ mice had a significant reduction in tumor growth and volume as compared to CCR5^+/+^ mice. The results are expressed as mean ± SD. * indicates significant difference from CCR5^+/+^ mice (P<0.05). **D**, The CCR5^−/−^ mice demonstrated significantly higher survival rates compared to CCR5^+/+^ mice (by Log-rank Test). **E**. Tumor sections were analyzed by H&E stain and expression of proliferating cell nuclear antigen (PCNA) by immunohistochemistry. Data are means ± S.D. of four experimental animals. * indicates significant difference from CCR5^+/+^ mice (P<0.05). Scale bar indicates 50 m.

The effect of CCR5 in tumor development was investigated using CCR5^+/+^ and CCR5^−/−^ mice. B16-F0 melanoma cells were inoculated subcutaneously into CCR5^+/+^ and CCR5^−/−^ mice (n = 20). Tumor growth was monitored for 31 days. Tumor growth in the CCR5^−/−^ mice was significantly lower than that in the CCR5^+/+^ mice. The tumor growth in the CCR5^−/−^ mice was reduced to 19.1%, whereas the tumor growth in the CCR5^+/+^ mice was only reduced to 24.3%, in both tumor weight (Figure 1A and 1B) and tumor volume, respectively (Figure 1A and 1C). These results correlated with mice survival rates. There was a significant difference in survival rates between CCR5^−/−^ mice (75% of starting time) and CCR5^+/+^ mice (15%) at the end of the experiment (Figure 1D). There was a significant difference in survival rates between CCR5^−/−^ mice and CCR5^+/+^ mice. At the end of the experiment, 75% of the CCR5^−/−^ mice survived while only 15% of the CCR5^+/+^ mice survived. Subcutaneous tumors were harvested from the sacrificed mice 31 days after inoculation. The immunohistochemical analysis of tumor sections, stained with H&E and for proliferation antigen PCNA, revealed greater inhibition of tumor cell growth in CCR5^−/−^ mice. The histologic findings after H&E staining indicated that the tumor tissues of the CCR5^−/−^ mice, but not those of the CCR5^+/+^ mice, contained large areas of necrosis. PCNA reactive cells were significantly reduced in the CCR5^−/−^ mice (Figure 1E).

### Decrease of NF-κB Activity in Tumor Tissue of CCR5^−/−^ Mice

**Figure 2:**
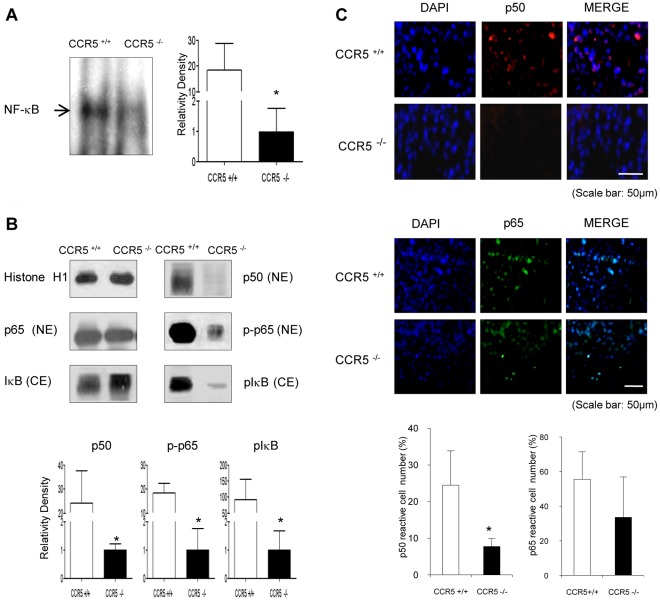
Decrease of NF-κB activity in tumor tissue of CCR5^−/−^ mice. **A**, The DNA binding activity of NF-κB was determined in the nuclear extracts of the CCR5^−/−^ mice and CCR5^+/+^ mice tumor tissues by EMSA described in material and methods. **B**, Expression of p50 and p65 phosphorylation in nuclear extracts (NE), and IκB and IκB phosphorylation in the cytosol (CE) determined by Western blotting. **C**, Immunolfluorescence analysis of p50 confirmed that the intensities of nuclear staining for p50 were decreased in the tumor tissues of CCR5^−/−^ mice. Data are means ± S.D. of four experimental animals. * indicates significant difference from CCR5^+/+^ mice (p<0.05). Scale bar indicates 50 µm.

The activation of NF-κB plays a critical role in cancer cell survival, especially melanoma cancer cells and in CCR5 expression. To evaluate whether the tumor inhibition was related with the inactivation of NF-κB in melanoma, the DNA binding activity of NF-κB was determined by electromobility shift assay (EMSA) in melanoma tissue. The constitutively activated DNA binding activity was reduced in the CCR5^−/−^ mice tumor tissue (Figure 2A). Moreover, the translocation of p50 and p-p65 into the nucleus through the inhibition of phosphorylation of IκB was also prevented in the tumor tissues of CCR5^−/−^ mice (Figure 2B). Immunofluorescence analysis also confirmed that the intensity of nuclear staining for p50 had decreased in the tumor tissues of CCR5^−/−^ mice (Figure 2C).

### Induction of Apoptotic Cell Death and Inhibition of the Expression of Anti-apoptotic Proteins in CCR5^−/−^ Mice

**Figure 3:**
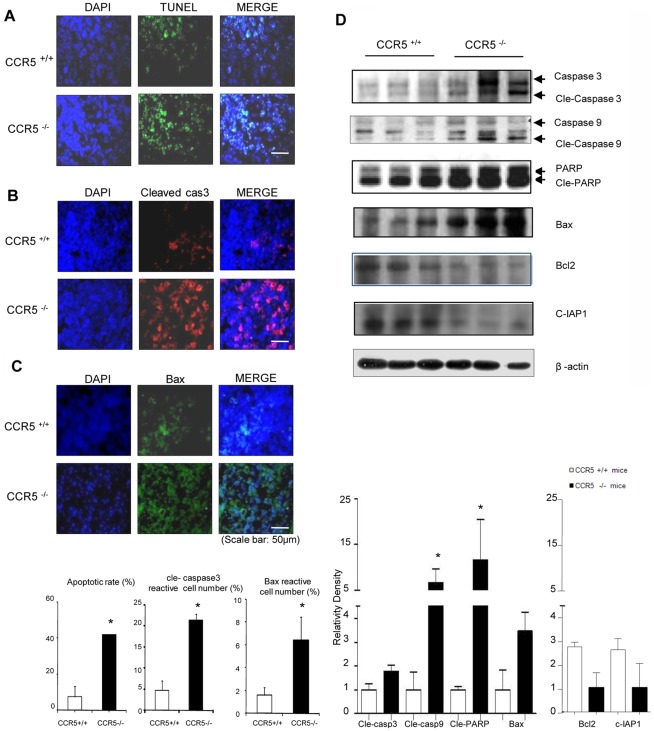
Induction of apoptotic cell death in tumor tissues of CCR5 ^−/−^ mice. **A**, Apoptotic cells in tumor sections were examined by fluorescence microscopy after DAPI and TUNEL staining as described in material and methods. The apoptotic index was determined as the number of DAPI-stained, TUNEL-positive cells that were counted. Values are the mean ± S.D. of four experimental animals. * indicates statistically significant differences from CCR5^+/+^ mice. Scale bar indicates 50 µm. **B and C**, Apoptotic protein (cleaved caspase-3 and Bax) in tumor sections were detected by immunofluorescence assay. The reactive cell number was determined as the number of DAPI-stained, Specific antibody (cleaved caspase-3 and Bax)-positive cells that were counted. Values are the mean ± S.D. of four experimental animals. * indicates statistically significant differences from CCR5^+/+^ mice. Scale bar indicates 50 µm. **D**, The expression of apoptotic proteins was detected by Western blotting using specific antibodies; cleaved caspase-3, cleaved caspase-9, cleaved PARP, Bax, Bcl-2 and c-IAP1 in the tumor tissues. The β-actin protein was used as an internal control. Each band is representative of three independent experimental results. Data are means ± S.D. of three experimental animals. * indicates significant difference from CCR5^+/+^ mice (p<0.05).

To see whether inhibition of NF-κB activity results in the induction of apoptotic cell death and expression of NF-κB target apoptotic cell death regulatory protein, the levels of apoptotic cell death and expression of apoptotic cell death regulatory proteins were assessed. Apoptotic cell death in the tumor sections were evaluated in the CCR5^+/+^ and CCR5^−/−^ mice that were inoculated with melanoma cells. TUNEL staining revealed a higher frequency of apoptotic cell death in the tumor tissues of the CCR5^−/−^ mice than that of the CCR5^+/+^ mice (Figure 3A). Immunohistochemical and immunofluorescence showed that high levels of cleaved caspase-3 and Bax were found more frequently in the CCR5^−/−^ mice than in the CCR5^+/+^ mice (Figure 3B and 3C). Consistent with the immunohistochemical data, Western blot analysis of tumor proteins showed greater increased levels of cleaved caspase-3, cleaved caspase-9, cleaved PARP and Bax, whereas the levels of Bcl-2 and C-IAP1 were significantly decreased in CCR5^−/−^ mice tissues (Figure 3D).

### Upregulation of IL-1Ra in the Tumor and Spleen of CCR5^−/−^ Mice

**Figure 4:**
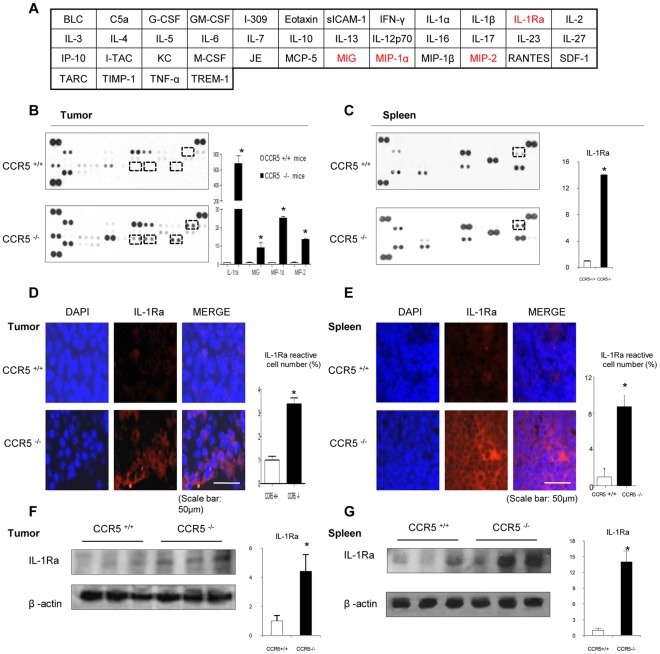
Upregulation of IL-1Ra in the tumor and spleen of CCR5^−/−^ mice. **A**, Figure 4A indicates mouse cytokine array panel coordinates. Nitrocellulose membranes contain 40 different anti-cytokine antibodies printed in duplicate. **B**, Mouse cytokine array panel indicate the cytokine expression difference in tumor tissues of CCR5^+/+^ mice and CCR5^−/−^ mice, especially IL-1Ra. Representative blot from three independent experiments is shown. Positive controls show the manufacturer’s internal positive control samples on the membrane. **C**, Protein immune-arrays were performed using Mouse cytokine array in spleen tissues. There were differences in cytokines between CCR5^+/+^ mice and CCR5^−/−^ mice, especially IL-1Ra. Representative blot from three independent experiments is shown. Positive controls show the manufacturer’s internal positive control samples on the membrane. **D and E**, Immunolfluorescence analysis was used to determine the expression levels of IL-1Ra in tumor and spleen tissues. The reactive cell number was determined as the number of DAPI-stained, IL-1Ra antibody-positive cells that were counted. Values are the mean ± S.D. of four experimental animals. * indicates statistically significant differences from CCR5^+/+^ mice. Scale bar indicates 50 µm. **F and G**, Expression of IL-1Ra was analyzed by western blotting in tumor and spleen tissues. Each band is representative of three independent experimental results. Data are means ± S.D. of three experimental animals. * indicates significant difference from CCR5^+/+^ mice (p<0.05).

To investigate the difference in the cytokine levels between the tumor and spleen tissues of the CCR5^+/+^ and CCR5^−/−^ mice, we conducted a cytokine array assay using a Mouse Proteome Array (Figure 4A). Among 40 tested cytokines, the levels of IL-1Ra, MIG, MIP-1α increased about 686, 9 and 25-fold respectively, in CCR5^−/−^ mice compared to CCR5^+/+^ mice. Using this technique, no other cytokines were found to be different between the CCR5^−/−^ mice and the CCR5^+/+^ mice (Figure 4B). Similar altered cytokine levels were found in the spleens of CCR5^−/−^ mice (Figure 4C). Since the IL-1Ra level was the most significantly elevated, the expression of IL-1Ra in tumor and spleen tissues was analyzed by immunofluorescence and western blotting. The expression of IL-1Ra was upregulated in tumor and spleen tissues of the CCR5^−/−^ mice (Figure 4D-G).

### Induction of CD8 T cells and NK Cell Infiltration into Tumor and Spleen of CCR5^−/−^ Mice

**Figure 5:**
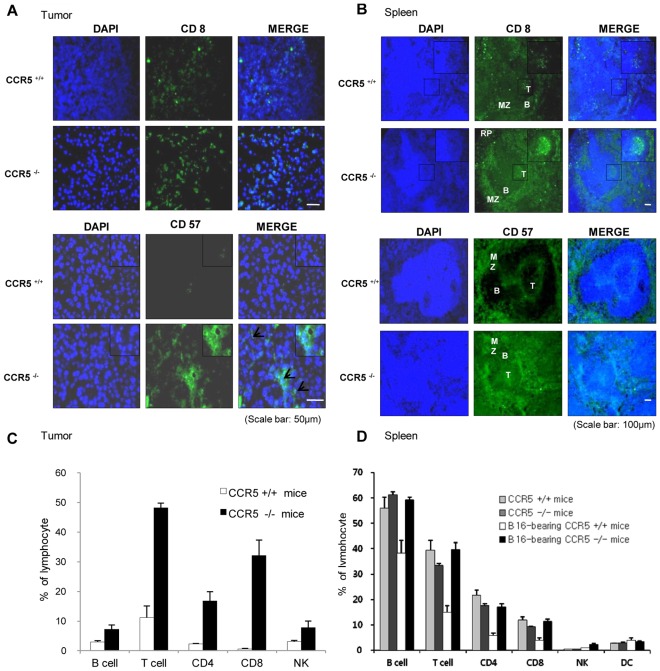
Induction of the infiltration of CD8^+^ T cells and NK cells into tumor, and increase in spleen of CCR5^−/−^ mice. **A and B**, Immunolfluorescence analysis was used to determine the expression levels of CD8 (CD8^+^ cytotoxic T-cell surface marker) and CD57 (NK cell marker) in tumor and spleen sections. The images shown are representative of three separate experiments performed in triplicate. Scale bars indicate 50 µm (A) and 100 µm (B). **C and D**, Analysis of lymphocyte phenotypes. The tumor and spleen tissues were separated at study termination (Day 31). Flow cytometry analysis was performed using FACSAria flow cytometry, and represent data were shown. Data are means ± S.D. of four experimental animals.

To investigate whether the inhibition of tumor growth in CCR5^−/−^ mice is related to tumor-specific immune responses, we analyzed the distribution patterns of CD8^+^ cytotoxic T cell and CD57^+^ Natural Killer cells in tumor and spleen tissues. By staining the tumor sections from CCR5^−/−^ mice, we found that there was a significant influx of CD8^+^ cells into the tumor, as well as an increase in the number of infiltrating NK cells (Figure 5A). CD8^+^ T cells and CD57^+^ NK cells were spread diffusely throughout the entire sections. To further investigate the differences in the numbers of CD8^+^ T cells and CD57^+^ NK cells between CCR5^+/+^ mice and CCR5^−/−^ mice in immunity-related organ, we also analyzed the CD8 and CD57 reactive cell number in spleen tissues. The number of CD8^+^ T cells and CD57^+^ NK cells were higher in the spleen of the CCR5^−/−^ mice than in the spleen of the CCR5^+/+^ mice (Figure 5B). These data suggest that by promoting the infiltration of CD8 T cells and NK cells, which are potent cytotoxic effectors for tumors, could play a role in an anti-tumor effect in CCR5^−/−^ mice.

To analyze the cell phenotype change in CCR5^−/−^ mice and CCR5^+/+^ mice by inoculation of the melanoma cell, lymphocytes were isolated from the tumor and spleen tissues. The B, CD3 T, CD4 T, CD 8 T and NK cell portion of CCR5^−/−^ mice tumor tissue were 7.2%, 48.2%, 16.9%, 32.1% and 7.8%, respectively, compared to 2.9%, 11.1%, 2.3%, 0.6% and 3.1% of CCR5^+/+^ mice (Figure 5C). The lymphocytes that infiltrated the tumor tissue of the CCR5^−/−^ mice also had higher levels of CD19, CD3, CD4, CD8 and CD57 when compared to the CCR5^+/+^ mice. The B, CD3 T, CD4 T, CD 8 T and NK cell portion of the CCR5^−/−^ mice spleen were 61.3%, 33.4%, 17.6%, 9.2% and 0.6%, respectively compared to 38.3%, 5.9%, 4.0%, 1.0% and 3.9% of the CCR5^+/+^ mice (Figure 5D). These data suggest that by promoting the infiltration of CD8 T cells and NK cells, which are potent cytotoxic effectors for tumors, could play a role in the inhibition of tumor growth in CCR5^−/−^ mice.

## Discussion

In the present study, we found that tumor weight and volume were much smaller in the CCR5^−/−^ mice compared to the CCR5^+/+^ mice inoculated with B16 melanoma cells (Figure 1A and 1B). The tumor growth inhibition in the CCR5^−/−^ mice was associated with the inhibition of constitutively activated NF-κB and elevation of IL-1Ra in the tumor tissue. This anti-tumor activity was also associated with decreased expression of NF-κB target anti-apoptotic, cell proliferative, and tumor promoting genes (Bcl-2 and C-IAP1) but with increased expression of their target apoptotic genes; cleaved caspase-3 and 9, and Bax (Figure 3A-D). In the tumor and the spleen, the number of cytotoxic CD8^+^ T cells, CD57^+^ natural killer cells, and the levels of IL-1Ra were increased (Figure 4 and 5). These findings suggest that melanoma growth was inhibited in the CCR5^−/−^ mice, and that the elevation of IL-1Ra, accompanied with decreased NF-κB, is significant in the inhibition of melanoma growth in CCR5^−/−^ mice.

NF-κB regulates the expression of over 200 genes that control the immune system, cancer cell growth and inflammation [39]. Because of its abilities to induce the expression of a large array of inflammatory mediators and its roles as core transcription factors in diverse immune responses, NF-κB has been recognized as a major factor responsible for cytokine-associated cancer development or anti-tumor immunity. In the melanoma tumor tissues of the CCR5^−/−^ mice, the expression of NF-κB target cell death genes (Bax, caspase-3 and 9) and the DNA binding activity of NF-κB, were significantly inhibited, but the expression of cell death inhibitory NF-κB target genes, such as Bcl-2 and cIAP were enhanced. In addition, CCR5^−/−^ prevented the phosphorylation of IκB, accompanied with the inhibition of the p50 and p65 translocation into the nucleus (Figure 2A-C). Many tumors, including melanoma, have increased levels of NF-κB [40], which is likely acting as a survival factor for melanoma growth. Thus, the inhibitions of NF-κB activity and the expression of target genes are critical in the inhibition of tumor growth in CCR5^−/−^ mice. Although the mechanism is not clear as to how CCR5^−/−^ downregulates NF-κB, it is noteworthy that NF-κB is activated or inactivated by many cytokines.

Numerous studies showed that cytokine affect tumor growth by regulating NF-κB. Inflammatory cytokines, including IL-1, IL-6 and IL-8, activate NF-κB pathways in tumor cells [41]. The interleukin 10 (IL-10) is well known as an anti-tumor factor as well as an anti-inflammatory factor [42], [43]. IL-10 inhibits constitutively activated NF-κB both *in vitro* [44] and *in vivo* [45]. Inhibition of NF-κB by the IL-10 contributes to the anti-tumor effects through the activation of caspase-3 and apoptosis in Colon26-bearing mice [43]. Similarly, several studies have demonstrated the ability of IL-1Ra to abrogate the proinflammatory effects of IL-1 [46]. It has been reported that IL-1 induces the activation of NF-κB in several cancers, and increases cell cycle progression, and the suppression of apoptotic cell death leading to tumor promotion. The results of various studies over the past 10 years indicate that the major function of IL-1Ra is to regulate the pleiotropic effects of IL-1 by competitively blocking its functions [47]. The ability of IL-1 to induce its own antagonist is of interest in understanding the regulation and dysregulation of IL-1 signaling in normal and diseased tissue. Up-regulation of the receptor antagonist represents a mechanism for turning off IL-1 signaling and creating a temporary state of refractoriness. The elevated expression of IL-1Ra, without any change of IL-1 in tumors, suggests that the IL-1-signaling system may be dysregulated by IL-1Ra in tumors. IL-1Ra has been shown to inhibit tumor progression in prostate, breast, and skin cancer [14], [15], [16]. Intracellular IL-1Ra (icIL-1Ra) inhibits IL-6 and IL-8 production in Caco-2 cells by blocking NF-κB pathways [37]. We found that the levels of IL-1Ra were significantly elevated in the tumor tissues of CCR5^−/−^ mice inoculated with B16 melanoma cells. Taken together, these data indicate IL-1Ra could be critically involved in the tumor growth inhibition of CCR5^−/−^ mice through the inactivation of NF-κB in the present study.

CCR5 increases tumor growth in a local model and inhibits the efficacy of a dendrite cell vaccine in CCR5^+/+^ mice compared with CCR5^−/−^ mice [10]. Cheng J and Sung RS examined the effect of CCR5 on the immune response to adenovirus vectors and graft function in an islet transplant model. They found that CCR5 absence does not prevent the local immune response to adenovirus transduction [48]. Additionally, Tan MC et al. suggested that disruption of CCR5/CCL5 signaling, either by reducing CCL5 production by tumor cells or by systemic administration of a CCR5 inhibitor (TAK-779), reduced Treg (regulatory T cells) migration to tumors so that tumors are smaller in control mice [9]. These data support the important premise that inhibition of tumor growth in CCR5^−/−^ mice could be related with the inhibition of immune escape. Similar to this finding, we found that the CCR5^+/+^ mice injected with melanoma cells apparently have a lower number of cytotoxic T cells (CD8+positive T Lymphocytes cells) and natural killer cells (CD57 positive NK cells), as well as dendrite cells, in spleen tissues compared to those without melanoma cells. But the lymphocytes of the CCR5^−/−^ mice did not decrease when injected with melanoma cells. These differences of the immune response between the CCR5^+/+^ mice and the CCR5^−/−^ mice, involving tumor immune tolerance, may be connected with the inhibition of cancer progression. Taken together, in the cytokine array experiment, the level of MIP-1α (which has a potent activity of T cell and B cell migration) was increased in the CCR5^−/−^ mice compared with those in the CCR5^+/+^ mice. Thus, relatively increased tumor associated cytotoxic lymphocytes could play an important role in inhibiting the tumor growth in CCR5^−/−^ mice.

Activation of NF-κB could modulate subcellular localization of NK cells and pathogenesis [49]. Inhibition of NF-κB activity prevented NK cell depletion, and thus increased anti-tumor activity by decreasing IL-6 production [50]. Thus, the decreased NF-κB and elevated IL-1Ra could also be associated in the infiltration of cytotoxic lymphocytes into tumor, resulting in tumor growth inhibition. In contrast, IL-23, a tumor growth promoting cytokine, increased NF-κB and immune cell infiltration in oral tumor [51]. These data indicate that NF-κB could be involved in the cytokine mediated anti-tumor activities of immune cells. It is also noteworthy that IL-1Ra inhibited melanoma tumor growth by increasing the number of myeloid suppressor cells in tumor [25]. The 3-methylcholatrene-induced tumor incidence was reduced in IL-1α knockout mice, but increased in IL-1Ra mice with concomitant maturation of NK cells and anti-tumor immunity [52]. These data indicate that the decrease of NF-κB, and thus increase of IL-1Ra could be significant in tumor growth inhibition of CCR5^−/−^ mice.

Our data thus show significant clinical implications of CCR5, because CCR5 is a G protein–coupled receptor and it is amenable to use as a small molecule for the inhibition of CCR5. Several such compounds have already been shown to be safe in clinical trials for use in certain diseases such as HIV [53]. Thus, we believe CCR5 is a cancer target that warrants continued investigation. In summary, this study showed that melanoma cells growth was inhibited via modulation of NF-κB/IL-1Ra pathways in CCR5^−/−^ mice.

## Materials and Methods

### Ethics Statement

All experiments were approved and carried out according to the Guide for the Care and Use of Animals [Animal Care Committee of Chungbuk National University, Korea (CBNUA-045-0902-01)].

### Animals

The CCR5 wild type (CCR5^+/+^) and CCR5 knockout (CCR5^−/−^) mice were purchased from The Jackson Laboratory (Bar Harbor, Maine 04609 USA). The mice were housed and bred under specific pathogen free conditions at the Laboratory Animal Research Center of Chungbuk National University, Korea. The mice (n = 4/cage) were maintained in a room with a constant temperature of 22±1°C, relative humidity of 55±10%, and 12-h light/dark cycle, and fed standard rodent chow (Samyang, Korea) and purified tap water *ad libitum*. The CCR5^+/+^ mice (n = 20) and CCR5^−/−^ mice (n = 20), that were used, had matched ages (10–11 weeks old) and weights (16–19 g). Control mice (B6129PF2/J) were F2 hybrid mice from the C57BL/6J-AW-J and 129P3/J parental strains. CCR5 mice (B6, 129P2-Ccr5tm1Kuz/J) have the entire coding region of the CCR5 gene deleted from the parental strains, B6;129P2-Ccr5tm1kuz and B6;129P2-Cmkbr5tm1Kuz.

### Tumor Inoculation and Tumor Monitoring

B16 melanoma cells were injected subcutaneously [5×10^5^ tumor cells in 0.1 ml Phosphate buffered saline(PBS)/animal] using a 27 G needle into the right-lower flanks of the carrier mice as previously described [54]. The body weights and tumor volumes of the animals were monitored twice weekly. The tumor volumes were measured with Vernier calipers and calculated using the following formula: (A×B^2^)/2, where A is the larger and B is the smaller of the two dimensions. At the end of the experiment, the animals were sacrificed. The tumors were separated from the surrounding muscles and dermis.

### Western Blotting

Tumor tissues were homogenized with protein extraction solution (PRO-PREPTM, Intron Biotechnology, Seoul, Korea), and lysed by 60 min incubation on ice. The tissue lysate centrifuged at 15,000 rpm for 15 min at 4°C. Equal amounts of proteins (40 µg) were separated on a SDS/12%-polyacrylamide gel, and then transferred to a polyvinylidene difluoride (PVDF) membrane (GE Water and Process technologies, Trevose, PA, USA). Blots were blocked for 1 h at room temperature with 5% (w/v) non-fat dried milk in Tris-Buffered Saline Tween-20 [TBST: 10 mM Tris (pH 8.0) and 150 mM NaCl solution containing 0.05% Tween-20]. After a short washing in TBST, the membranes were immunoblotted with the following primary antibodies: mouse monoclonal antibodies directed against p65 and p50 (1∶500 dilutions; Santa Cruz Biotechnology, Santa Cruz, CA), rabbit polyclonal antibodies directed against Bax and PARP (1∶500 dilutions; Santa Cruz Biotechnology), and against caspase-3, caspase-9, Bcl-2 and c-IAP-1 (1∶1000 dilutions; Cell Signaling Technology, Beverly, MA). The blots were incubated with the respective horseradish peroxidase-conjugated anti-rabbit or anti-mouse IgG (1∶4000 dilutions; Santa Cruz Biotechnology). Immunoreactive proteins were detected with the ECL detection system.

### Immunohistochemistry

All specimens were fixed in formalin and embedded in paraffin for examination. Sections (4 µm thick) were stained with H&E and analyzed by immunohistochemistry. The paraffin-embedded sections were deparaffinized and rehydrated, washed in distilled water, and then subjected to heat-mediated antigen retrieval. Endogenous peroxidase activity was quenched by incubation in 3% hydrogen peroxide in methanol for 15 min, followed by clearing in PBS for 5 min. The sections were blocked for 30 min with 3% normal horse serum diluted in PBS, blotted, and incubated with primary mouse anti-mouse proliferating cell nuclear antigen (PCNA, 1∶200 dilution) monoclonal antibodies in blocking serum for 4 h at room temperature. Thereafter, the slides were washed three times for 5 min each in PBS and incubated with biotinylated anti-mouse and anti-rabbit antibodies for 2 h. The slides were washed in PBS, followed by the addition of the avidin biotin peroxidase complex (ABC kit; Vector Laboratories, Burlingame, CA, USA). The slides were washed and the peroxidase reaction was developed with diaminobenzidine (DAB) and peroxide, followed by counterstaining with hematoxylin, mounting in aqua-mount, and evaluation under a light microscope (magnification×200; Olympus, Tokyo, Japan). A negative control was included in all experiments by omitting the primary antibody. For the detection of apoptotic cell death in the tumor tissues, the paraffin-embedded sections were incubated in a mixture of the labeling solution (540 µl) and enzyme solution (60 µl) for 1 h at 37°C, and then washed three times in 0.1 M PBS for 5 min each, according to the manufacturer’s instructions. Next, the sections were incubated with 4′,6-diamidino-2-phenylindole (DAPI) for 15 min at 37°C. Finally, the sections were rinsed, mounted on slides, and cover-slipped for fluorescence microscopy (DAS microscope).

### Immunofluorescence

Sections were treated with 10% bovine serum albumin in PBS for 1 h at room temperature, incubated overnight at 4°C with p50 (1∶250, Santa Cruz Biotechnology, Santa Cruz, CA), p65 (1∶250, Santa Cruz Biotechnology, Santa Cruz, CA), Bax (1∶200 dilution), cleaved caspase-3 (1∶100), CD8 (1∶10), CD57 (1∶50) and IL-1Ra (1∶500) antibodies, followed by incubation in anti-mouse IgG conjugated with Alexa 488 (1∶100 dilution, Molecular Probes, Eugene, OR, USA) and anti-rabbit IgG conjugated with Alexa 568 (1∶100 dilution, Molecular Probes) for 40 min at room temperature. Finally, the sections were rinsed, mounted on slides, and cover-slipped for fluorescence microscopy and photography using ApoTome microscopy (Carl Zeiss, Thornwood, NY, USA).

### Gel Electromobility Shift Assay

A gel electromobility shift assay (EMSA) was performed according to the manufacturer’s recommendations (Promega, Madison, WI). The tumor tissues were briefly homogenized in 200 µl of solution A (10 mM HEPES [pH 7.9], 1.5 mM MgCl_2_, 10 mM KCl, 0.5 mM dithiothreitol, 0.2 mM phenylmethylsulfonylfluoride), vortexed vigorously, incubated on ice for 10 min, and then centrifuged at 15000 rpm for 15 min. The pelleted nuclei were resuspended in solution C (solution A supplemented with 420 mM NaCl and 20% glycerol), and incubated on ice for 20 min. The resuspended pellet was centrifuged at 15000 rpm for 15 min, and the resulting nuclear extracts supernatant were collected in a chilled Eppendorf tube. Consensus oligonucleotides were end-labeled using T4 polynucleotide kinase and [P^32^]-ATP for 10 min at 37°C. The gel shift reactions were assembled and incubated at room temperature. Subsequently, 1 ml of gel loading buffer was added to each reaction and loaded onto a 6% non-denaturating gel. The gel was subjected to electrophoresis until the dye was four-fifths of the way down the gel. The gel was dried for 1 h at 80°C and exposed to film overnight at -70°C.

### Flow Cytometric Analysis

Lymphocytes were obtained from tumor and spleen tissues for phenotype analysis. Spleen and tumor lymphocytes were isolated from fresh spleen and tumor biopsies obtained from three mice per group. Spleen and tumor biopsies were briefly homogenized mechanically in PBS, filtered (100 µm cell strainer; BD PharMingen) and then placed in lysis buffer (ACK lysing buffer, LONZA) to remove red blood cells. One million splenocytes were washed once in PBS containing 1% bovine serum albumin (BSA) and re-suspended in 100 µl of PBS/BSA buffer. Meanwhile, one million cells obtained from tumor tissues were placed in RPMI medium, incubated to obtain suspension cell for 1hour at 37°C, centrifuged (1200 rpm, 3 min) and then washed in PBS. Next, the lymphocytes were incubated with various conjugated monoclonal antibodies, including CD4-APC, CD8-FITC and CD19-PE for 20min at 4°C, washed twice in PBS, and re-suspended in 400 µl of PBS. A flow cytometric analysis was performed on a FACSAria flow cytometry (BD Biosciences), and the data were analyzed using the WinMDI statistical software (Scripps, La Jolla, CA, USA). Forward and side scatter parameters were used to gate on lymphocytes.

### Protein Immuno-arrays

The tumor and spleen tissues were excised and homogenized in PBS with protease inhibitor cocktail (Sigma-Aldrich, USA) and Triton X-100 (final concentration 1%). The samples were frozen at −70*°*C, thawed, and centrifuged at 10,000×g for 5 min to remove cellular debris. The tissue lysates were performed protein assay in the same way as for western blotting. 4.5 mg of proteins, collected from three samples per group, were used for Mouse cytokine array as the protocol provided by supplier (Proteome Profiler™, #ARY006, R&D Systems, USA).

### Statistical Analysis

The data were analyzed using the GraphPad Prism 4 ver. 4.03 software (GraphPad Software, La Jolla, CA). Data are presented as mean ± SD. Difference between CCR5^−/−^ mice and CCR5^+/+^ mice were compared using *t* test. A value of *p*<0.05 was considered to be statistically significant. Survival data were presented by Kaplan-Meier survival estimates and compared and calculated by Log-rank (Mantel-Cox) Test in GraphPad Prism.
